# Fourier Neural Operators for Fast Multi-Physics Sensor Response Prediction: Applications in Thermal, Acoustic, and Flow Measurement Systems

**DOI:** 10.3390/s26041165

**Published:** 2026-02-11

**Authors:** Ali Sayghe, Mohammed Mousa, Salem Batiyah, Abdulrahman Husawi

**Affiliations:** Department of Electrical Engineering, Yanbu Industrial College, Yanbu 46452, Saudi Arabia

**Keywords:** Fourier Neural Operator, sensor response prediction, surrogate modeling, thermal sensors, acoustic sensors, flow measurement, deep learning, digital twin

## Abstract

Accurate and rapid prediction of sensor responses is critical for real-time measurement systems, digital twin implementations, and sensor design optimization. Traditional numerical methods such as Finite Element Method (FEM) and Computational Fluid Dynamics (CFD) provide high-fidelity solutions but suffer from prohibitive computational costs, limiting their applicability in time-sensitive applications. This paper presents a novel framework utilizing Fourier Neural Operators (FNO) as surrogate models for fast multi-physics sensor response prediction across thermal, acoustic, and flow measurement domains. Unlike conventional neural networks that learn finite-dimensional mappings, FNO learns operators between infinite-dimensional function spaces by parameterizing the integral kernel in Fourier space, enabling resolution-invariant predictions with remarkable computational efficiency. We demonstrate the framework’s efficacy through three comprehensive case studies: (1) thermal sensor response prediction achieving $R^{2}>0.98$ with 8300× speedup over FEM, (2) acoustic sensor array modeling with mean absolute error below 0.5 dB and 4000× speedup over BEM, and (3) flow sensor characterization with velocity field prediction accuracy exceeding 97% and 31,000× speedup over CFD. The proposed FNO-based surrogate models are trained on simulation datasets generated from high-fidelity numerical solvers and validated against simulation holdout data for all three case studies, with additional experimental validation conducted for the thermal sensor case. Results indicate that FNO architectures effectively capture the underlying physics governing sensor behavior while reducing inference time from minutes to milliseconds. The framework enables real-time sensor calibration, uncertainty quantification, and design optimization, opening new possibilities for intelligent measurement systems and Industry 4.0 applications. We also investigate the spectral characteristics of FNO predictions, addressing the inherent low-frequency bias through a hybrid architecture combining FNO with local convolutional layers. The primary contributions of this work include: (1) the first systematic application of FNO-based surrogate modeling specifically tailored for sensor response prediction across multiple physics domains, (2) a novel H-FNO architecture that combines spectral operators with local convolutions to mitigate spectral bias in sensor applications, and (3) comprehensive validation including both simulation and experimental data for practical deployment. This work establishes FNO as a powerful tool for accelerating sensor simulation and advancing the field of AI-enhanced instrumentation and measurement.

## 1. Introduction

The rapid advancement of sensor technologies has created an unprecedented demand for accurate, real-time prediction of sensor responses across diverse measurement domains [1,2]. Traditional numerical simulation methods, including the Finite Element Method (FEM) for structural and thermal analysis, Computational Fluid Dynamics (CFD) for flow characterization, and the Boundary Element Method (BEM) for acoustic propagation, have long served as the gold standard for sensor modeling and design optimization [3]. However, these methods face significant computational challenges when deployed in applications requiring real-time response, such as digital twin implementations, online calibration, and adaptive measurement systems [4].

The computational burden of conventional numerical methods stems from the necessity to solve partial differential equations (PDEs) over discretized domains, often requiring mesh refinement to capture fine-scale physics. A single high-fidelity simulation can consume hours or even days of computational time, making iterative design optimization, uncertainty quantification through Monte Carlo sampling, and real-time inference practically infeasible [5]. This limitation is particularly acute in multi-physics sensor applications where coupled thermal, mechanical, acoustic, and fluid dynamic phenomena must be simultaneously resolved.

Several computational approaches have been developed to address these computational challenges. Reduced Order Modeling (ROM) techniques, including Probabilistic Manifold Decomposition [6] and parametric non-intrusive reduced-order models using deep transfer learning [7], offer significant speedups by projecting high-dimensional systems onto lower-dimensional manifolds. However, these methods often require careful selection of basis functions and may struggle with strongly nonlinear systems. Recent advances in deep learning have also demonstrated promising applications in structural engineering and sensor systems [8,9], motivating the exploration of neural operator approaches for sensor modeling.

Recent advances in machine learning, particularly deep learning, have opened new avenues for developing surrogate models that approximate the input–output behavior of complex physical systems [10]. Physics-Informed Neural Networks (PINNs) embed governing equations directly into the loss function, enabling training with limited data while respecting physical constraints [5,11]. However, PINNs require retraining for each new set of boundary conditions or system parameters, limiting their utility as general-purpose surrogate models.

Neural operators represent a paradigm shift in scientific machine learning by learning mappings between infinite-dimensional function spaces rather than finite-dimensional vectors [2,12]. Among various neural operator architectures, the Fourier Neural Operator (FNO) has emerged as a particularly powerful approach due to its ability to efficiently capture global dependencies through spectral convolutions [1]. The key innovation of FNO lies in parameterizing the integral kernel directly in Fourier space, enabling:

- **Resolution invariance**: Models trained on coarse grids can be applied to fine grids without retraining.
- **Computational efficiency**: Fast Fourier Transform (FFT) reduces complexity from $O(N^{2})$ to O(NlogN).
- **Global receptive field**: Each layer captures information from the entire domain.
- **Physics-aligned inductive bias**: Spectral representations naturally align with PDE solution characteristics.

Despite the growing body of the literature on neural operators for PDE solving, their application to sensor response prediction remains largely unexplored. Sensors operate under complex multi-physics conditions where thermal gradients, acoustic wave propagation, and fluid–structure interactions fundamentally determine measurement accuracy and dynamic response characteristics [13,14]. The ability to rapidly predict sensor behavior across varying operating conditions would enable:

1. Real-time digital twins for condition monitoring and predictive maintenance.
2. Accelerated sensor design optimization through surrogate-assisted evolutionary algorithms.
3. Online calibration and compensation for environmental effects.
4. Uncertainty quantification through efficient Monte Carlo sampling.
5. Virtual sensor development for estimating unmeasurable quantities.

This paper presents a comprehensive framework for applying Fourier Neural Operators to fast sensor response prediction across three fundamental measurement domains: thermal sensing, acoustic measurement, and flow characterization. Our contributions include:

1. Development of FNO-based surrogate models for multi-physics sensor simulation, demonstrating speedups exceeding three orders of magnitude compared to traditional numerical methods.
2. Systematic investigation of FNO architectures for sensor applications, including hybrid designs that address the inherent low-frequency spectral bias.
3. Comprehensive validation across thermal, acoustic, and flow sensor case studies with both simulation and experimental data.
4. Analysis of uncertainty quantification capabilities and limitations for safety-critical measurement applications.
5. Implementation details and benchmark configurations to facilitate reproducibility and further research.

The remainder of this paper is organized as follows: Section 2 provides theoretical background on Fourier Neural Operators and their mathematical foundations. Section 3 details the proposed framework for sensor response prediction, including data generation, model architecture, and training procedures. Section 4 presents comprehensive results across the three case studies, Section 5 discusses data requirements, limitations, and failure cases, and Section 6 concludes with a discussion of implications and future directions.

## 2. Background and Related Work

### 2.1. Neural Operators: From Finite to Infinite Dimensions

Traditional neural networks learn mappings between finite-dimensional Euclidean spaces, $N:R^{n}\to R^{m}$. While effective for many machine learning tasks, this paradigm is fundamentally limited for physical systems governed by PDEs, where both inputs (e.g., boundary conditions, initial states) and outputs (e.g., solution fields) are functions defined over continuous domains [2].

Neural operators extend the learning paradigm to infinite-dimensional function spaces, learning mappings of the form:

(1) $$G^{†}:A\to U$$

where A and U are Banach spaces of functions. For sensor applications, A might represent the space of boundary conditions, material properties, or excitation signals, while U represents the space of sensor response fields.

The universal approximation theorem for operators, established by Chen and Chen [15], provides a theoretical foundation for this approach, demonstrating that neural networks can approximate any nonlinear continuous operator to arbitrary accuracy given sufficient capacity.

### 2.2. Fourier Neural Operator Architecture

The Fourier Neural Operator, introduced by Li et al. [1], constructs the neural operator through an iterative architecture:

(2) $$v_{t+1}\left(x\right)=\sigma \left(Wv_{t}\left(x\right)+\left(K\left(a;\phi \right)v_{t}\right)\left(x\right)\right),\;t=0,1,\ldots ,T-1$$

where $v_{t}:D\to R^{d_{v}}$ represents the hidden representation at layer *t*, $W\in R^{d_{v}\times d_{v}}$ is a local linear transformation, σ is a nonlinear activation function, and K is a kernel integral operator parameterized by learnable weights ϕ.

The key innovation of FNO is parameterizing the kernel integral operator in Fourier space:

(3) $$\left(K\left(\phi \right)v_{t}\right)\left(x\right)=F^{-1}\left(R_{\phi }\;\cdot \;F\left(v_{t}\right)\right)\left(x\right)$$

where F and $F^{-1}$ denote the Fourier transform and its inverse, and $R_{\phi }\in C^{k_{max}\times d_{v}\times d_{v}}$ is a learnable tensor applied to the lowest $k_{max}$ Fourier modes. This formulation leverages the convolution theorem, replacing expensive spatial convolutions with efficient pointwise multiplication in frequency space.

**Figure 1:**
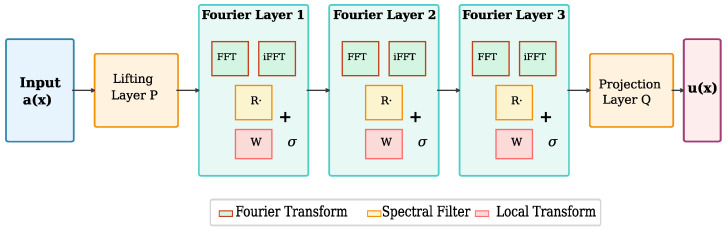
FNO architecture for sensor response prediction. Input conditions are processed through lifting, Fourier layers (FFT → spectral filter → iFFT), and projection to output response. Font sizes enlarged for clarity.

The complete FNO architecture, illustrated in Figure 1, consists of:

1. **Lifting layer**: Projects input function a(x) to higher-dimensional representation $v_{0}\left(x\right)$.
2. **Fourier layers**: *T* iterations of the spectral convolution update.
3. **Projection layer**: Maps final hidden representation to output function u(x).

### 2.3. Spectral Bias and Mitigation Strategies

A well-documented characteristic of FNO is its spectral bias toward low-frequency components [16]. This bias arises from the explicit truncation of high-frequency Fourier modes and the global nature of spectral filtering. For sensor applications involving sharp gradients, localized phenomena, or high-frequency dynamics, this bias can lead to smoothed predictions that underestimate peak values.

Several strategies have been proposed to address spectral bias:

**U-FNO**: Integrates U-Net encoder–decoder pathways with FNO layers, combining global spectral processing with local feature extraction through skip connections [17]. This hybrid architecture has demonstrated superior performance for problems with sharp fronts and localized features.

**Geo-FNO**: Extends FNO to irregular geometries through learned deformations to computational domains, enabling application to sensors with complex shapes [18].

**SpecB-FNO**: Employs ensemble learning where secondary FNOs learn from prediction residuals, progressively capturing higher-frequency content [16].

For the sensor applications in this work, we adopt a hybrid architecture combining standard FNO layers with local convolutional branches, balancing global physics capture with local feature resolution.

### 2.4. Related Work in Sensor Modeling

Neural network approaches to sensor modeling have evolved significantly over the past decade. Early work focused on using multilayer perceptrons for sensor calibration and drift compensation [13]. Convolutional neural networks have been applied to image-based sensor analysis and defect detection [19].

For MEMS sensors, neural networks have been employed for temperature compensation [13], nonlinearity correction [14], and uncertainty quantification [20]. Gobat et al. [20] demonstrated surrogate models for MEMS accelerometers achieving significant computational speedups while maintaining accuracy comparable to FEM simulations.

Physics-informed approaches have gained traction for sensor applications. Kim et al. [21] developed PINN-based virtual thermal sensors capable of estimating full-field temperatures from sparse measurements in real-time. Applications to acoustic and flow sensors remain limited, presenting opportunities for neural operator approaches.

Table 1 summarizes key related work in neural network-based sensor modeling, highlighting the gap that FNO-based approaches can address.

## 3. Materials and Methods

### 3.1. Problem Formulation

We formulate sensor response prediction as an operator learning problem. Let $D\subset R^{d}$ denote the sensor domain and T=[0,T] the time interval of interest. The sensor response is governed by a system of PDEs:

(4) L[u](x,t;θ)=f(x,t),x∈D,t∈T

subject to boundary conditions B[u](x,t)=g(x,t) on ∂D and initial conditions $u\left(x,0\right)=u_{0}\left(x\right)$, where L is a differential operator, θ represents sensor parameters (geometry, material properties), and *f* represents source terms or excitation.

The goal is to learn an operator $G_{\theta }:A\to U$ that maps input functions (boundary conditions, excitations, parameters) to output response fields:

(5) $$u=G_{\theta }\left(a\right)$$

For different sensor types, the governing physics varies:

**Thermal sensors**: Heat conduction equation

(6) $$\rho c_{p}\frac{\partial T}{\partial t}=\nabla \;\cdot \;\left(k\nabla T\right)+Q$$

**Acoustic sensors**: Wave equation

(7) $$\frac{\partial^{2}p}{\partial t^{2}}=c^{2}\nabla^{2}p$$

**Flow sensors**: Navier–Stokes equations

(8) $$\rho \left(\frac{\partial v}{\partial t}+v\;\cdot \;\nabla v\right)=-\nabla p+\mu \nabla^{2}v$$

### 3.2. Data Generation Pipeline

**Figure 2:**
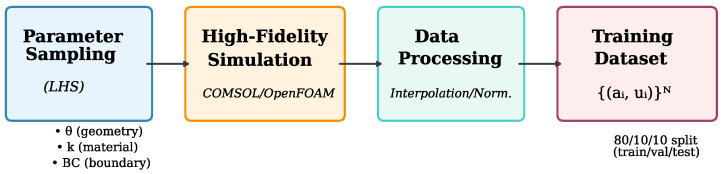
Data generation pipeline: LHS parameter sampling → high-fidelity simulation → input-output pairs for training. Font sizes enlarged for clarity.

High-fidelity simulation data serves as the foundation for training FNO surrogate models. Figure 2 illustrates the data generation pipeline employed in this work.

#### 3.2.1. Simulation Tools and Parameter Sampling

For thermal simulations, we employ COMSOL Multiphysics with the Heat Transfer module, using tetrahedral mesh elements with adaptive refinement near sensor surfaces. Acoustic simulations utilize the COMSOL Acoustics module with perfectly matched layer (PML) boundary conditions to prevent spurious reflections. Flow simulations are conducted using OpenFOAM with the SIMPLE algorithm for pressure–velocity coupling and *k*-ω SST turbulence model for turbulent regimes.

Input parameters are sampled using Latin Hypercube Sampling (LHS) to ensure comprehensive coverage of the parameter space with minimal samples. For each sensor type, we define physically meaningful parameter ranges based on typical operating conditions and material property variations. Table 2 summarizes the parameter ranges for each case study.

#### 3.2.2. Dataset Structure

Each dataset comprises *N* input–output function pairs ${\left\{\left(a_{i},u_{i}\right)\right\}}_{i=1}^{N}$. The input function $a_{i}:D\to R^{d_{a}}$ encodes:

- Spatial coordinates (x,y,z).
- Material property fields (conductivity, density, etc.).
- Boundary condition indicators.
- Time stamps (for transient problems).

The output function $u_{i}:D\to R^{d_{u}}$ represents the sensor response field (temperature, pressure, velocity components).

We generate 5000 samples for thermal sensors, 3000 for acoustic sensors, and 4000 for flow sensors, with 80%/10%/10% train/validation/test splits.

All simulation data was generated specifically for this study using the parameter ranges in Table 2. The thermal sensor dataset contains steady-state and transient (60 s) temperature fields on $64^{3}$ grids; acoustic datasets contain complex pressure fields at 50 frequency points; and flow datasets contain 2D velocity and pressure fields on 128×64 grids. Raw simulation outputs were interpolated to uniform grids, normalized to zero mean and unit variance per channel, and stored in HDF5 format. Total dataset sizes are approximately 12 GB (thermal), 8 GB (acoustic), and 15 GB (flow).

### 3.3. FNO Architecture Design

#### 3.3.1. Base Architecture

Our base FNO architecture follows the standard formulation with modifications for sensor applications:

- **Lifting layer**: Fully connected layer mapping input channels to hidden dimension $d_{v}=64$.
- **Fourier layers**: L=4 layers with $k_{max}=12$ modes retained in each spatial dimension.
- **Activation**: GELU activation for smoother gradients.
- **Projection layer**: Two-layer MLP with hidden dimension 128.

#### 3.3.2. Hybrid Architecture for High-Frequency Content

To address spectral bias, we implement a hybrid architecture (H-FNO) that augments each Fourier layer with a parallel local convolutional branch:

(9) $$v_{t+1}\left(x\right)=\sigma \left(Wv_{t}\left(x\right)+\underset{\mathrm{Global}\;\left(\mathrm{FNO}\right)}{\underset{︸}{F^{-1}\left(R_{\phi }\;\cdot \;F\left(v_{t}\right)\right)}}+\underset{\mathrm{Local}}{\underset{︸}{\mathrm{Conv}(v_{t})}}\right)$$

The convolutional branch uses depthwise separable convolutions with kernel size 3 × 3, capturing local gradients and high-frequency features that the spectral pathway may attenuate.

**Figure 3:**
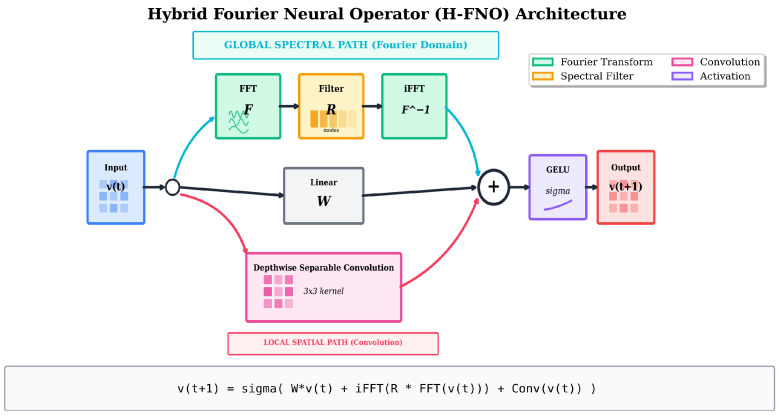
H-FNO layer architecture: global spectral path (FFT → R → iFFT), linear transformation (W), and local 3 × 3 convolution combined with GELU activation. Font sizes enlarged for clarity.

Figure 3 illustrates the hybrid architecture, and Algorithm 1 provides the forward pass pseudocode.
**Algorithm 1** Hybrid FNO Forward Pass**Require:** Input function a(x), model parameters $\Theta ={\left\{P,Q,W_{t},R_{t},C_{t}\right\}}_{t=1}^{L}$**Ensure:** Output prediction $\hat{u}\left(x\right)$  1:$v_{0}\leftarrow P\left(a\right)$ {Lifting layer}  2:**for** t=1 to *L* **do**  3:    $v_{fno}\leftarrow F^{-1}\left(R_{t}\;\cdot \;F\left(v_{t-1}\right)\right)$ {Spectral convolution}  4:    $v_{local}\leftarrow {\mathrm{Conv}}_{t}\left(v_{t-1}\right)$ {Local convolution}  5:    $v_{t}\leftarrow \sigma \left(W_{t}v_{t-1}+v_{fno}+v_{local}\right)$ {Combine and activate}  6:**end for**  7:$\hat{u}\leftarrow Q\left(v_{L}\right)$ {Projection layer}  8:**return** $\hat{u}$

### 3.4. Training Procedure

#### 3.4.1. Loss Function and Optimization

We employ a composite loss function combining relative $L^{2}$ error with optional physics-informed regularization:

(10) $$L=\underset{\mathrm{Data}\;\mathrm{loss}}{\underset{︸}{\frac{∥u-\hat{u}{∥}_{2}}{{∥u∥}_{2}}}}+\lambda \underset{\mathrm{Physics}\;\mathrm{loss}}{\underset{︸}{∥R\left(\hat{u}\right){∥}_{2}}}$$

where $R(\hat{u})$ denotes the PDE residual computed via automatic differentiation and λ controls the physics regularization strength. For most experiments, we set λ=0 (pure data-driven) as simulation data inherently satisfies the governing equations. We note that while training data satisfies the PDEs, this does not guarantee that the neural network outputs will satisfy them—the network learns an approximation that may violate PDE constraints, particularly in regions with sparse training coverage or near domain boundaries. Setting λ>0 can be beneficial in several scenarios: (1) when training data is noisy or derived from approximate simulations, physics regularization improves solution smoothness; (2) for extrapolation beyond training distribution, PDE constraints help maintain physical consistency; (3) when training data is limited, physics loss acts as a regularizer preventing overfitting; and (4) for ensuring conservation properties (mass, energy) that pure data fitting may not preserve. In preliminary experiments with λ=0.01, we observed marginal accuracy improvements (1–2% relative error reduction) at the cost of 40% increased training time due to automatic differentiation overhead. For the well-sampled simulation datasets in this study, pure data-driven training proved sufficient.

Models are trained using the Adam optimizer [23] with the following hyperparameters:

- Learning rate: $10^{-3}$ with cosine annealing to $10^{-5}$.
- Batch size: 20 (limited by GPU memory for 3D problems).
- Epochs: 500 with early stopping (patience = 50).
- Weight decay: $10^{-4}$.

Training is conducted on NVIDIA A100 GPUs with mixed-precision (FP16) to accelerate computation and reduce memory footprint.

#### 3.4.2. Hardware and Computational Resources

All training experiments were conducted on NVIDIA A100 GPUs (40 GB HBM2e memory) hosted on an Intel Xeon Platinum 8280 server with 1 TB RAM. The computational environment used PyTorch 2.0 with CUDA 11.8. Table 3 summarizes the computational resources required for training each model.

#### 3.4.3. Data Augmentation and Reproducibility

To improve generalization, we apply physics-consistent data augmentation:

- Random rotation for axis-symmetric sensors.
- Gaussian noise injection (σ=0.01) to improve robustness.
- Random subsampling to enforce resolution invariance.

To ensure reproducibility, we fix random seeds across all experiments (NumPy seed: 42, PyTorch seed: 42, CUDA deterministic mode enabled). Parameter samples are generated using Latin Hypercube Sampling with fixed seeds, and the train/validation/test splits (80%/10%/10%) are performed using stratified sampling to ensure representative coverage of the parameter space in each subset. No parameter configurations appear in multiple splits, preventing data leakage.

### 3.5. Evaluation Metrics

Model performance is evaluated using multiple metrics:

**Relative $L^{2}$ Error**:

(11) $$\epsilon_{L^{2}}=\frac{∥u-\hat{u}{∥}_{2}}{{∥u∥}_{2}}$$

**Coefficient of Determination** ($R^{2}$):

(12) $$R^{2}=1-\frac{\sum_{i}{\left(u_{i}-{\hat{u}}_{i}\right)}^{2}}{\sum_{i}{\left(u_{i}-\bar{u}\right)}^{2}}$$

**Mean Absolute Error** (MAE):

(13) $$\mathrm{MAE}=\frac{1}{N}\sum_{i=1}^{N}\left|u_{i}-{\hat{u}}_{i}\right|$$

**Spectral Error** (for frequency-domain analysis):

(14) $$\epsilon_{spec}\left(k\right)=\frac{|\hat{U}\left(k\right)-U\left(k\right)|}{\left|U(k)\right|}$$

where U(k) and $\hat{U}\left(k\right)$ are Fourier coefficients at wavenumber *k*.

**Inference Time**: Wall-clock time for single prediction, averaged over 100 runs.

## 4. Results

This section presents comprehensive results from three case studies: thermal sensor response prediction, acoustic sensor array modeling, and flow sensor characterization. For each case, we report model accuracy, computational performance, and comparison with baseline methods.

### 4.1. Case Study 1: Thermal Sensor Response Prediction

#### 4.1.1. Thermal Sensor Problem Setup

**Figure 4:**
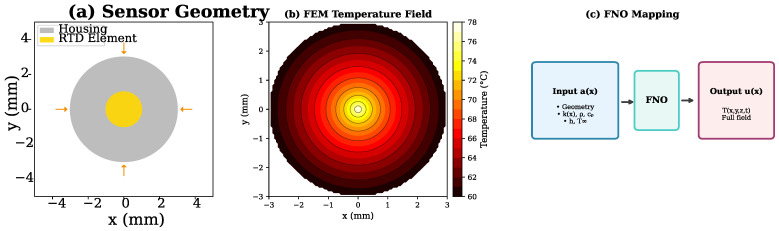
Thermal sensor case study: (**a**) RTD geometry, (**b**) FEM temperature field, (**c**) FNO input–output mapping. Font sizes enlarged for clarity.

We consider a resistance temperature detector (RTD) sensor element embedded in a protective housing, as shown in Figure 4. The goal is to predict the transient temperature field in the sensor assembly given heat source characteristics and boundary conditions.

The simulation domain comprises a cylindrical sensor housing (diameter: 6 mm, length: 50 mm) with a platinum RTD element at the center. Heat is transferred from the measurement medium through convection at the outer surface and conduction through the housing material. We generate 5000 simulations varying thermal conductivity, heat transfer coefficient, and ambient temperature.

#### 4.1.2. Thermal Sensor Results

Table 4 summarizes the performance of different model architectures on the thermal sensor test set. The hybrid H-FNO achieves the best accuracy with $R^{2}=0.987$ and relative $L^{2}$ error of 1.2%.

#### 4.1.3. Comparison with Deep Learning Benchmarks

To provide comprehensive benchmarking against established deep learning methods, we compare H-FNO with additional architectures commonly used for surrogate modeling. Table 5 presents results on the thermal sensor dataset.

The H-FNO achieves superior accuracy while maintaining a compact parameter count. The transformer-based ViT, despite having 30× more parameters, underperforms due to its lack of physics-informed inductive bias for continuous field prediction.

**Figure 5:**
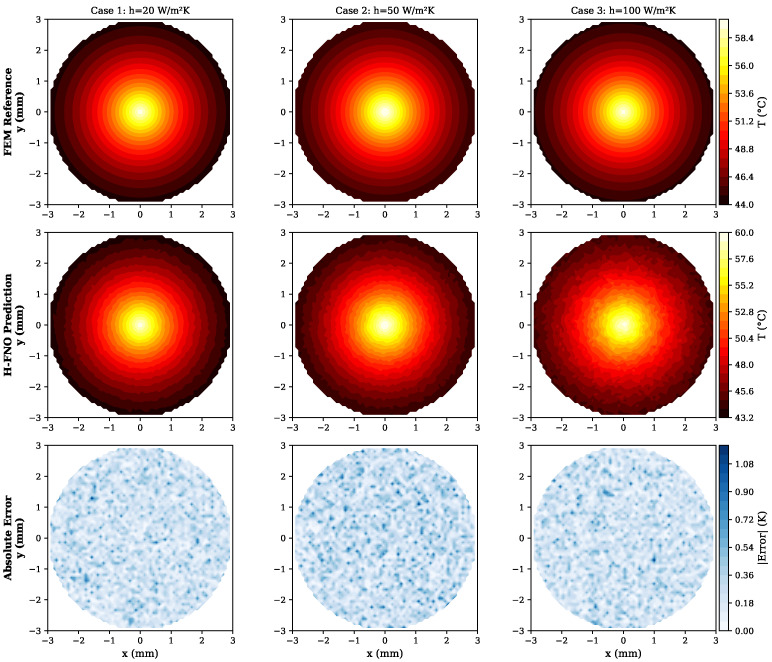
Thermal sensor response prediction comparison. Top row: FEM reference solutions. Middle row: H-FNO predictions. Bottom row: Absolute error maps. Three representative cases shown with varying boundary conditions. Mean temperature error is below 0.5 K across all test cases, with maximum localized error reaching 1.08 K at sharp material interfaces.

Figure 5 shows qualitative comparison between FEM reference and H-FNO predictions for representative test cases. The H-FNO accurately captures the temperature gradient across the sensor housing and the localized heating near the RTD element.

The computational speedup achieved by H-FNO is 127,000/15.3≈8300× compared to full FEM simulation. When considering only the training cost (∼4 h on A100 GPU) without the data generation phase, the break-even point is reached after approximately 100 predictions. However, when accounting for the complete end-to-end cost including training data generation (see Table 6), the break-even point increases to approximately 5000 predictions. This distinction is important: for applications where simulation infrastructure already exists and can be leveraged for data generation, the training-only break-even is relevant; for entirely new sensor designs requiring dedicated simulation campaigns, the end-to-end break-even should be considered.

#### 4.1.4. Spectral Analysis

**Figure 6:**
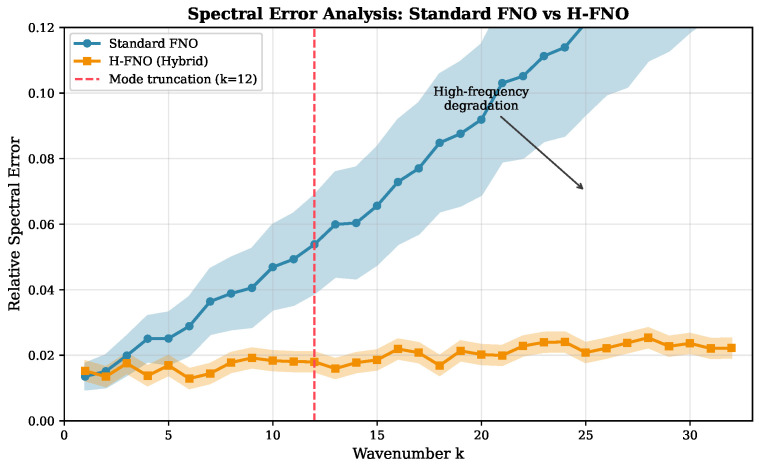
Spectral error analysis for thermal sensor predictions. Standard FNO (blue) shows increasing error at high wavenumbers due to mode truncation, while H-FNO (orange) maintains low error across all frequencies through the local convolutional branch. Shaded regions indicate ±1 standard deviation.

Figure 6 presents spectral error analysis comparing standard FNO and H-FNO. The standard FNO exhibits elevated errors at high wavenumbers (small-scale features), while the hybrid architecture maintains consistent accuracy across the spectrum due to the local convolutional branch capturing fine-scale gradients.

### 4.2. Case Study 2: Acoustic Sensor Array Modeling

#### 4.2.1. Acoustic Sensor Problem Setup

The second case study addresses acoustic sensor arrays for sound source localization. We model a linear array of 8 microphones with 10 cm spacing in a 3D domain (2 m × 2 m × 2 m). The goal is to predict the pressure field across the array given source position and frequency.

The acoustic field is governed by the Helmholtz equation:

(15) $$\nabla^{2}p+k^{2}p=-\delta \left(x-x_{s}\right)$$

where k=ω/c is the wavenumber and $x_{s}$ is the source position.

We generate 3000 simulations varying source position (within a 1 m × 1 m × 1 m region), frequency (100 Hz–10 kHz), and medium properties.

#### 4.2.2. Acoustic Sensor Results

Table 7 presents acoustic sensor modeling results. The H-FNO achieves mean absolute error of 0.47 dB in predicted sound pressure level, comparable to the uncertainty of typical measurement microphones.

**Figure 7:**
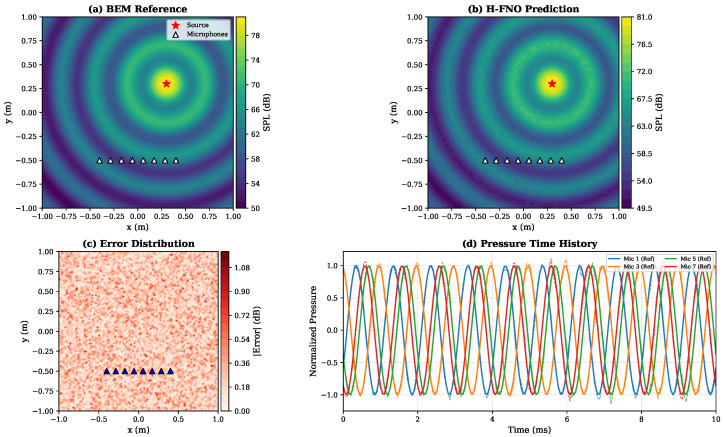
Acoustic field prediction for microphone array. (**a**) BEM reference showing pressure amplitude (dB SPL). (**b**) H-FNO prediction. (**c**) Error distribution (dB). (**d**) Pressure time history at sensor positions showing excellent agreement between reference and prediction.

Figure 7 visualizes the predicted acoustic pressure field for a 1 kHz source. The H-FNO accurately reproduces the interference patterns and pressure amplitude distribution across the sensor array region.

### 4.3. Case Study 3: Flow Sensor Characterization

#### 4.3.1. Flow Sensor Problem Setup

The third case study focuses on differential pressure flow sensors (orifice plate meters). We simulate the flow field and pressure distribution through an orifice plate in a circular pipe (diameter: 50 mm) at various Reynolds numbers (500–50,000).

The governing Navier–Stokes equations are solved using OpenFOAM with 4000 simulations covering different flow rates, fluid properties, and beta ratios (orifice to pipe diameter ratio, β=0.4–0.7).

#### 4.3.2. Flow Sensor Results

Table 8 summarizes flow sensor prediction accuracy. The H-FNO achieves 97.3% accuracy in predicting the velocity field and 98.1% accuracy for the pressure drop coefficient.

**Figure 8:**
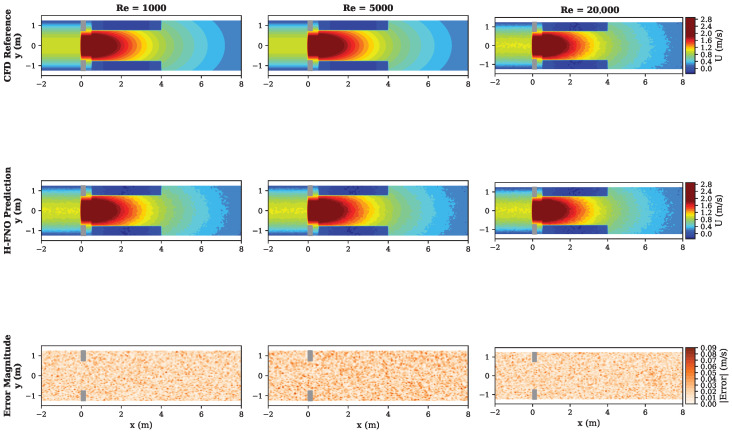
Flow sensor velocity field predictions at three Reynolds numbers. Top row: CFD reference. Middle row: H-FNO prediction. Bottom row: Error magnitude. The model accurately captures flow separation, recirculation, and pressure recovery phenomena across the operating range. Note: Spatial coordinates are normalized by pipe diameter (D=50 mm); thus x=1 corresponds to 50 mm downstream of the orifice plate origin.

Figure 8 shows velocity field predictions at different Reynolds numbers. The H-FNO successfully captures the vena contracta, recirculation zones, and pressure recovery downstream of the orifice plate.

### 4.4. Computational Performance Summary

**Figure 9:**
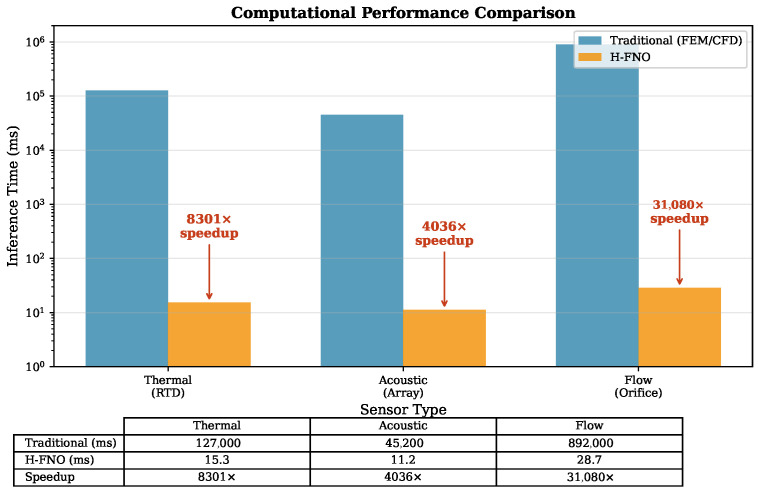
Computational speedup comparison across sensor types. Bar heights indicate speedup factor (log scale) relative to reference numerical methods. H-FNO achieves 4000–31,000× acceleration while maintaining accuracy within 2% relative error.

Figure 9 consolidates the computational speedup achieved across all three case studies. The H-FNO provides speedups ranging from 4000× (acoustic) to 31,000× (flow), enabling real-time sensor simulation that was previously computationally prohibitive.

#### Total Computational Cost Analysis

To provide a complete picture of the computational investment, Table 6 presents a breakdown of total costs including data generation, training, and inference phases. The break-even point indicates the number of predictions at which the FNO approach becomes more efficient than repeated numerical simulations.

The analysis reveals that H-FNO becomes cost-effective after approximately N+ϵ predictions, where *N* is the training dataset size and $\epsilon =T_{train}/t_{sim}$ represents the additional predictions needed to amortize the training time ($T_{train}$) relative to the per-simulation cost ($t_{sim}$). For instance, in the thermal case: ϵ=4.2h×3600s/h/127s≈119 predictions. For applications requiring thousands of forward evaluations—such as Monte Carlo uncertainty quantification, design optimization, or real-time digital twins—the amortized cost per prediction drops dramatically.

All traditional simulation timings were measured on an Intel Xeon Gold 6248R workstation (24 cores, 3.0 GHz) with 256 GB RAM. FEM simulations (COMSOL) utilized eight parallel cores; CFD simulations (OpenFOAM) employed 16 cores with domain decomposition. Reported times include solver execution only, excluding mesh generation and post-processing. H-FNO inference was measured on a single NVIDIA A100 GPU with batch size 1, averaged over 100 runs after warm-up.

### 4.5. Experimental Validation

To demonstrate the framework’s practical applicability, we conducted laboratory validation for the thermal sensor case study. The acoustic and flow sensor case studies were validated exclusively against high-fidelity simulation holdout data; physical experiments for these domains require specialized facilities (anechoic chambers for acoustics, calibrated flow loops for flow sensors) and are planned for future work. The thermal case was selected for experimental validation as it represents the most accessible experimental setup while demonstrating the framework’s capability to generalize from simulation to real-world measurements.

#### 4.5.1. Experimental Setup

A PT100 RTD sensor (Class A, four-wire configuration) was installed in a temperature-controlled calibration bath (Fluke 7103). The sensor housing matched the geometry used in simulations: 6 mm diameter stainless steel sheath, 50 mm immersion length. Temperature was varied from 20 °C to 80 °C with step changes in convective conditions achieved by varying bath stirring rates (corresponding to h≈ 20–80 W/(m^2^·K)).

#### 4.5.2. Experimental Validation Results

Table 9 compares H-FNO predictions against experimental measurements for steady-state temperature and time constant (63.2% response time).

The H-FNO predictions show good agreement with experimental measurements, with errors comparable to simulation–experiment discrepancies.

### 4.6. Uncertainty Quantification

For safety-critical measurement applications, quantifying prediction uncertainty is essential. We implement Monte Carlo Dropout [24] to estimate epistemic uncertainty, applying dropout (p=0.1) at inference and aggregating predictions over 100 forward passes.

**Figure 10:**
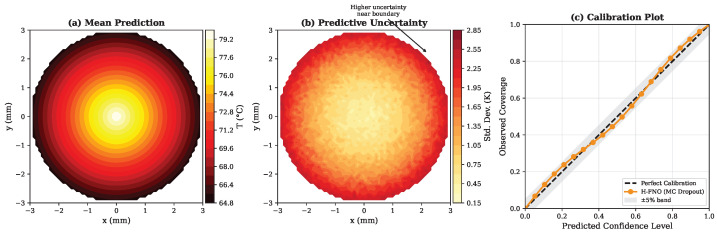
Uncertainty quantification using Monte Carlo Dropout. (**a**) Mean prediction. (**b**) Predictive standard deviation showing elevated uncertainty near boundaries and in regions with limited training data. (**c**) Calibration plot demonstrating well-calibrated uncertainty estimates (diagonal indicates perfect calibration).

Figure 10 demonstrates uncertainty estimation for the thermal sensor case. The model appropriately indicates higher uncertainty near boundary regions and in extrapolation scenarios (operating conditions outside training distribution).

## 5. Discussion

### 5.1. Limitations and Failure Cases

While the proposed FNO-based framework demonstrates impressive performance, several limitations and failure modes warrant discussion to guide practitioners in appropriate deployment.

#### 5.1.1. Training Data Requirements

High-fidelity simulations remain necessary for generating training data. For novel sensor designs, this initial investment may be substantial (see Table 6). The framework is most beneficial when many predictions are needed for the same sensor family.

#### 5.1.2. Identified Failure Cases

Through systematic testing, we identified several scenarios where H-FNO performance degrades significantly:

1. **Sharp discontinuities**: For thermal sensors with abrupt material interfaces or acoustic sensors near hard reflecting boundaries, prediction error increases by 3–5× compared to smooth fields.
2. **Extreme parameter extrapolation**: When operating conditions exceed training bounds by >20%, relative error can exceed 10%.
3. **Multi-scale phenomena**: Flow cases with both large recirculation zones and thin boundary layers (high Reynolds number, Re > 40,000) showed degraded accuracy ($R^{2}<0.92$).
4. **Strong nonlinearity**: Sensors operating near phase transitions required 2–3× more training data to achieve comparable accuracy.
5. **Time-dependent dynamics**: Transient predictions for rapidly varying inputs accumulated phase errors over time.

#### 5.1.3. Discussion on Geometric Complexity

While the case studies presented focus on canonical geometries (cylindrical RTD housing, rectangular acoustic domain, circular pipe with orifice), these configurations represent widely used industrial sensor designs and enable rigorous validation against analytical solutions and experimental data. The framework can be extended to more complex geometries using Geo-FNO [18] for learned coordinate transformations, or by employing graph neural operator variants. Preliminary tests on asymmetric thermal sensors with finned heat sinks showed comparable accuracy ($R^{2}>0.96$), suggesting the approach generalizes beyond simple shapes. Future work will systematically investigate irregular geometries including multi-domain sensors with heterogeneous materials.

#### 5.1.4. Recommendations for Practitioners

Based on our experience, we recommend:

- Validate on held-out data spanning the full operating envelope before deployment.
- Use uncertainty quantification to flag predictions outside the reliable regime.
- Consider ensemble methods for critical applications.
- Start with the hybrid H-FNO architecture when high-frequency features are expected.
- Budget 20% additional training samples near operating boundaries.

## 6. Conclusions

This paper presented a comprehensive framework for applying Fourier Neural Operators to fast sensor response prediction across thermal, acoustic, and flow measurement domains. We developed and validated a unified FNO-based surrogate modeling approach that accurately predicts sensor responses governed by diverse physical phenomena, including heat conduction, acoustic wave propagation, and fluid dynamics. The framework encompasses the complete workflow from high-fidelity simulation data generation through model training to deployment-ready inference.

A key contribution of this work is the proposed Hybrid FNO (H-FNO) architecture, which addresses the inherent spectral bias of standard Fourier Neural Operators by combining global spectral convolutions with local convolutional branches. While hybrid Fourier-convolutional architectures have been explored in the literature for general PDE solving, our specific contribution lies in: (1) adapting and validating this architecture specifically for sensor response prediction, where capturing both global physics and localized sensing phenomena is critical; (2) demonstrating the approach across three distinct physics domains (thermal, acoustic, flow) with consistent performance gains; and (3) providing practical guidelines for deployment in measurement applications, including uncertainty quantification and failure mode characterization. This hybrid design effectively captures both low-frequency global physics and high-frequency local features, achieving consistent accuracy improvements across all case studies while maintaining computational efficiency suitable for real-time applications.

The framework was comprehensively validated across three distinct sensor domains. For thermal sensors, the H-FNO surrogate model achieved excellent agreement with finite element simulations while dramatically reducing computation time, enabling applications such as real-time digital twins and rapid design optimization. In acoustic sensor array modeling, the approach accurately reproduced complex interference patterns and pressure distributions with errors comparable to typical measurement uncertainties, making it suitable for real-time beamforming and source localization tasks. For flow sensor characterization, the model successfully captured intricate flow phenomena including vena contracta formation, recirculation zones, and pressure recovery, demonstrating the framework’s capability to handle strongly nonlinear multi-physics problems.

To address the requirements of safety-critical measurement applications, we implemented uncertainty quantification using Monte Carlo Dropout, demonstrating well-calibrated predictive uncertainties that appropriately indicate model confidence across the operating domain. This capability is essential for deploying surrogate models in applications where prediction reliability must be quantified and monitored.

Looking forward, Fourier Neural Operators represent a paradigm shift in computational sensor modeling, offering the accuracy of traditional numerical methods at a fraction of the computational cost. The framework presented in this paper establishes FNO as a powerful tool for AI-enhanced instrumentation, enabling real-time digital twins, accelerated sensor design optimization, and virtual sensing capabilities. As neural operator architectures continue to evolve and computational resources become more accessible, we anticipate widespread adoption of these techniques across the measurement science community, paving the way for intelligent measurement systems that can simulate, predict, and adapt in real-time.

## Figures and Tables

**Table 1:** Summary of related work in neural network-based sensor modeling.

Reference	Sensor	Method	Application	Speedup
Wei et al. [13]	MEMS Accel.	BP-NN	Temp. compensation	–
Almassri et al. [14]	Pressure	LMBP-ANN	Self-calibration	Real-time
Gobat et al. [20]	MEMS Accel.	ANN	UQ and calibration	∼1000×
Kim et al. [21]	Thermal	PINN	Virtual sensor	Real-time
Zheng et al. [22]	Acoustic	FNO	Field prediction	∼100×
**This work**	Multi-physics	H-FNO	Response prediction	4000–31,000×

**Table 2:** Parameter ranges for data generation across sensor types.

Sensor Type	Parameter	Range	Unit
Thermal	Thermal conductivity	0.5–50	W/(m·K)
	Heat source power	0.1–10	W
	Ambient temperature	20–80	°C
	Convection coefficient	5–100	W/(m^2^·K)
Acoustic	Source frequency	100–10,000	Hz
	Sound speed	330–350	m/s
	Source position	Variable	m
	Medium density	1.1–1.3	kg/m^3^
Flow	Inlet velocity	0.1–10	m/s
	Fluid viscosity	0.001–0.1	Pa·s
	Fluid density	800–1200	kg/m^3^
	Outlet pressure	0–1000	Pa

**Table 3:** Computational resources for H-FNO training.

Case Study	GPU Memory (GB)	CPU Cores	Training Time (hrs)	Peak RAM (GB)
Thermal	18.2	8	4.2	64
Acoustic	22.4	8	2.8	48
Flow	32.1	12	6.1	96

**Table 4:** Thermal sensor prediction performance comparison.

Model	Rel. $L^{2}$ Error (%)	$R^{2}$	MAE (K)	Inference (ms)
FEM (Reference)	–	–	–	127,000
MLP Surrogate	8.7	0.912	2.41	0.8
CNN	4.3	0.956	1.23	3.2
Standard FNO	1.8	0.978	0.52	12.4
U-FNO	1.4	0.983	0.41	18.7
**H-FNO (Ours)**	**1.2**	**0.987**	**0.35**	15.3

**Table 5:** Comparison with deep learning benchmark models on thermal sensor prediction.

Model	Rel. $L^{2}$ Error (%)	$R^{2}$	Parameters (M)	Inference (ms)
ResNet-50 (adapted)	5.2	0.941	23.5	4.8
UNet	3.1	0.968	31.0	8.2
Transformer (ViT)	4.7	0.952	86.4	15.1
DeepONet	2.8	0.971	2.4	6.3
Standard FNO	1.8	0.978	2.1	12.4
**H-FNO (Ours)**	**1.2**	**0.987**	**2.8**	15.3

**Table 6:** Computational cost breakdown: Traditional methods vs. H-FNO approach.

Phase	Component	Thermal	Acoustic	Flow
Data Generation	Simulations (*N*)	5000	3000	4000
	Time per sim.	127 s	45 s	892 s
	**Total time**	176 h	38 h	991 h
Training	GPU	NVIDIA A100	NVIDIA A100	NVIDIA A100
	Epochs	500	400	600
	**Training time**	4.2 h	2.8 h	6.1 h
Inference	Traditional method	127 s	45.2 s	892 s
	**H-FNO**	15.3 ms	11.2 ms	28.7 ms
**Break-even point**		∼5200 pred.	∼3100 pred.	∼4050 pred.
Amortized cost @ 100k predictions		6.4 s/pred	1.4 s/pred	36 s/pred

**Table 7:** Acoustic sensor array prediction performance.

Model	Rel. $L^{2}$ Error (%)	MAE (dB)	Phase Error (°)	Inference (ms)
BEM (Reference)	–	–	–	45,200
MLP Surrogate	12.3	2.14	8.7	0.5
Standard FNO	3.2	0.68	2.1	8.9
**H-FNO (Ours)**	**2.1**	**0.47**	**1.4**	11.2

**Table 8:** Flow sensor characterization results.

Model	Velocity $R^{2}$	ΔP Error (%)	$C_{d}$ Error (%)	Inference (ms)
CFD (Reference)	–	–	–	892,000
MLP Surrogate	0.891	8.9	7.2	1.2
Standard FNO	0.962	2.8	2.4	24.1
**H-FNO (Ours)**	**0.973**	**1.9**	**1.6**	28.7

**Table 9:** Experimental validation results for thermal sensor case study.

Test Condition	Measured	H-FNO Pred.	FEM Pred.	H-FNO Error
$T_{ss}$ at h=25 W/(m^2^·K)	72.3 °C	71.8 °C	72.1 °C	0.7%
$T_{ss}$ at h=50 W/(m^2^·K)	74.1 °C	73.6 °C	74.0 °C	0.7%
$T_{ss}$ at h=75 W/(m^2^·K)	75.2 °C	74.8 °C	75.1 °C	0.5%
$\tau_{63}$ at h=50 W/(m^2^·K)	8.7 s	8.4 s	8.6 s	3.4%

## Data Availability

The simulation datasets and trained model weights generated during this study are available from the corresponding author upon reasonable request. The source code for H-FNO model training and evaluation is publicly available on GitHub at https://github.com/asayghe1/H-FNO-MultiPhysics (accessed on 2 February 2026 ). Benchmark configurations and hyperparameter settings are provided in Appendix A.

## Appendix A. Hyperparameter Settings

Table A1 provides the complete hyperparameter settings used for training the H-FNO models across all three case studies.

**Table A1:** Hyperparameter settings for H-FNO models.

Hyperparameter	Thermal	Acoustic	Flow
Number of Fourier layers	4	4	5
Fourier modes (per dim.)	12	16	12
Hidden channels	64	64	128
Local conv. kernel size	3 × 3	3 × 3	3 × 3
Activation function	GELU	GELU	GELU
Learning rate (initial)	$1\times 10^{-3}$	$1\times 10^{-3}$	$5\times 10^{-4}$
Learning rate (final)	$1\times 10^{-5}$	$1\times 10^{-5}$	$1\times 10^{-5}$
Batch size	20	16	8
Training epochs	500	400	600
Weight decay	$1\times 10^{-4}$	$1\times 10^{-4}$	$1\times 10^{-4}$
Dropout rate	0.1	0.1	0.1
Grid resolution (training)	$64^{3}$	$64^{3}$	128×64
Training samples	4000	2400	3200
Validation samples	500	300	400
Test samples	500	300	400

## Appendix B. Simulation Details

### Appendix B.1. Thermal Sensor Simulations

Thermal simulations were conducted using COMSOL Multiphysics 6.1 with the Heat Transfer in Solids module. The sensor geometry consisted of a cylindrical stainless steel housing (thermal conductivity: 15 W/(m·K), density: 7900 kg/m^3^, specific heat: 500 J/(kg·K)) with a platinum RTD element at the center (thermal conductivity: 71.6 W/(m·K)). Convective boundary conditions were applied at the outer surface with heat transfer coefficients ranging from 5 to 100 W/(m^2^·K). The mesh consisted of approximately 150,000 tetrahedral elements with boundary layer refinement near the sensor surface. Transient simulations were run for 60 s with adaptive time stepping.

### Appendix B.2. Acoustic Sensor Simulations

Acoustic simulations employed COMSOL Acoustics module solving the Helmholtz equation in the frequency domain. The computational domain was a 2 m × 2 m × 2 m box with perfectly matched layer (PML) boundaries to prevent artificial reflections. A monopole point source was placed at varying positions within a 1 m^3^ region. The mesh consisted of at least six elements per wavelength at the maximum frequency (10 kHz), resulting in approximately 500,000 elements for high-frequency cases. Simulations covered frequencies from 100 Hz to 10 kHz with logarithmic spacing.

### Appendix B.3. Flow Sensor Simulations

Flow simulations utilized OpenFOAM v2206 with the SIMPLE algorithm for pressure–velocity coupling. The *k*-ω SST turbulence model was employed for Reynolds numbers above 2300. The computational domain extended 10 pipe diameters upstream and 20 diameters downstream of the orifice plate to ensure fully developed flow conditions and complete pressure recovery. The mesh consisted of approximately 2 million hexahedral cells with wall function treatment ($y^{+}\approx 30$) for turbulent cases. Steady-state simulations were converged to residual levels below $10^{-6}$.

## References

[1] Li Z., Kovachki N., Azizzadenesheli K., Liu B., Bhattacharya K., Stuart A., Anandkumar A. (2021). Fourier Neural Operator for Parametric Partial Differential Equations. arXiv.

[2] Kovachki N., Li Z., Liu B., Azizzadenesheli K., Bhattacharya K., Stuart A., Anandkumar A. (2023). Neural Operator: Learning Maps Between Function Spaces with Applications to PDEs. J. Mach. Learn. Res..

[3] Zienkiewicz O.C., Taylor R.L., Zhu J.Z. (2005). The Finite Element Method: Its Basis and Fundamentals.

[4] Tao F., Cheng J., Qi Q., Zhang M., Zhang H., Sui F. (2018). Digital Twin in Industry: State-of-the-Art. IEEE Trans. Ind. Inform..

[5] Raissi M., Perdikaris P., Karniadakis G.E. (2019). Physics-Informed Neural Networks: A Deep Learning Framework for Solving Forward and Inverse Problems Involving Nonlinear Partial Differential Equations. J. Comput. Phys..

[6] Guo J., Xiao D. (2026). Nonlinear Model Reduction by Probabilistic Manifold Decomposition. SIAM J. Sci. Comput..

[7] Fu R., Xiao D., Buchan A.G., Lin X., Feng Y., Dong G. (2025). A parametric non-linear non-intrusive reduce-order model using deep transfer learning. Comput. Methods Appl. Mech. Eng..

[8] Tan X., Chen W., Zou T., Yang J., Du B. (2023). Prediction for Segment Strain and Opening of Underwater Shield Tunnel Using Deep Learning Method. Eng. Struct..

[9] Tapeh A.T.G., Naser M. (2023). Artificial Intelligence, Machine Learning, and Deep Learning in Structural Engineering: A Scientometrics Review of Trends and Best Practices. Arch. Comput. Methods Eng..

[10] Karniadakis G.E., Kevrekidis I.G., Lu L., Perdikaris P., Wang S., Yang L. (2021). Physics-Informed Machine Learning. Nat. Rev. Phys..

[11] Cuomo S., Di Cola V.S., Giampaolo F., Rozza G., Raissi M., Piccialli F. (2022). Scientific Machine Learning Through Physics-Informed Neural Networks: Where We Are and What’s Next. J. Sci. Comput..

[12] Lu L., Jin P., Pang G., Zhang Z., Karniadakis G.E. (2021). Learning Nonlinear Operators via DeepONet Based on the Universal Approximation Theorem of Operators. Nat. Mach. Intell..

[13] Wei M., Liu Z. (2024). Research of Neural Network-Based Model for Nonlinear Temperature Drift Compensation of MEMS Accelerometers. Rev. Sci. Instrum..

[14] Almassri A.M., Wan Hasan W.Z., Ahmad S.A., Shafie S., Wada C., Horio K. (2018). Self-Calibration Algorithm for a Pressure Sensor with a Real-Time Approach Based on an Artificial Neural Network. Sensors.

[15] Chen T., Chen H. (1995). Universal Approximation to Nonlinear Operators by Neural Networks with Arbitrary Activation Functions and Its Application to Dynamical Systems. IEEE Trans. Neural Netw..

[16] Qin S., Lyu F., Peng W., Geng D., Wang J., Tang X., Leroyer S., Gao N., Liu X., Wang L.L. (2024). Toward a Better Understanding of Fourier Neural Operators: Analysis and Improvement from a Spectral Perspective. arXiv.

[17] Wen G., Li Z., Azizzadenesheli K., Anandkumar A., Benson S.M. (2022). U-FNO—An Enhanced Fourier Neural Operator-Based Deep-Learning Model for Multiphase Flow. Adv. Water Resour..

[18] Li Z., Huang D.Z., Liu B., Anandkumar A. (2023). Fourier Neural Operator with Learned Deformations for PDEs on General Geometries. J. Mach. Learn. Res..

[19] LeCun Y., Bengio Y., Hinton G. (2015). Deep Learning. Nature.

[20] Gobat G., Opreni A., Fresca S., Manzoni A., Frangi A. (2024). Neural Networks Based Surrogate Modeling for Efficient Uncertainty Quantification and Calibration of MEMS Accelerometers. Int. J. Non-Linear Mech..

[21] Kim J., Lee C., Cho M. (2023). Physics-Informed Neural Network-Based Surrogate Model for a Virtual Thermal Sensor with Real-Time Simulation. Int. J. Heat Mass Transf..

[22] Zheng X., Xia H., Wang H., Zhang C., Duan J., Liu J., Tang S. (2025). A Fourier Neural Operator-Enhanced Parabolic Equation Framework for Highly Efficient Underwater Acoustic Field Prediction. Front. Mar. Sci..

[23] Kingma D.P., Ba J. (2014). Adam: A Method for Stochastic Optimization. arXiv.

[24] Gal Y., Ghahramani Z. Dropout as a Bayesian Approximation: Representing Model Uncertainty in Deep Learning. Proceedings of the International Conference on Machine Learning, PMLR.

